# PRIMARY BONE LYMPHOMAS: RETROSPECTIVE ANALYSIS OF 42 CONSECUTIVE CASES

**DOI:** 10.1590/1413-785220182602185549

**Published:** 2018

**Authors:** TELMA MURIAS DOS SANTOS, JUAN PABLO ZUMÁRRAGA, FÁBIO MAZETTI REAES, CARLOS HENRIQUE MAÇANEIRO, ANDRÉ MATHIAS BAPTISTA, OLAVO PIRES DE CAMARGO

**Affiliations:** 1. Orthopedic Oncology Group, Instituto de Ortopedia e Traumatologia, Hospital das Clinicas HCFMUSP, Faculdade de Medicina, Universidade de São Paulo, São Paulo, SP, Brazil.; 2. Department of Orthopedics and Traumatology, Faculdade de Medicina, Universidade de São Paulo, São Paulo, SP, Brazil.

**Keywords:** Lymphoma/physiopathology, Lymphoma/therapy, Lymphoma, large B-cell, diffuse, Bone neoplasms, Drug therapy., Linfoma/fisiopatologia, Linfoma/terapia, Linfoma difuso de grandes células B, Neoplasias ósseas, Tratamento farmacológico.

## Abstract

**Objective:**

It is difficult to define parameters for management and factors associated with primary bone lymphoma (PBL). This article presents the experience in a single institution with 42 patients with PBL over a 16-year period (2000-2016).

**Methods:**

Fifty-five patients were retrospectively evaluated, and forty-two were included (76.3%).

**Results:**

Median age at diagnosis was 51.5 years, and median follow-up was 102.7 months. One patient had HIV. Pain in the affected site was the most prevalent symptom. The average time between symptom onset and diagnosis was 5.4 months. The vertebrae were most affected (n=16, 33.3%). According to the International Prognostic Index Score (IPI), 64.3% of the patients were classified as having low-grade lymphoma and 25.7% as low-intermediate. The most common histology was diffuse large B cell lymphoma (DLBCL) (85.7%). Immunophenotyping was CD20 positive in 93.5% of patients, and 11 patients had pathological fracture. All patients received chemotherapy and 30% of the regimens included rituximab. Thirty-eight percent of patients received radiation therapy. Overall survival was 50%, and survival median time was 80 months. Age and chemotherapy regimen influenced patient survival. Younger patients and patients who received RCHOP had better prognoses.

**Conclusions:**

The choice of chemotherapy regimen associated with age influenced survival for patients with PBL. Level of Evidence IV; Case series.

## RESUMO

**Objetivo:**

É difícil definir parâmetros para o tratamento e os fatores associados ao prognóstico de linfoma ósseo primário (LOP). Apresentamos a experiência de uma única instituição, com 42 casos de LOP durante 16 anos (2000-2016).

**Métodos:**

Cinquenta e cinco pacientes avaliados retrospectivamente e quarenta e dois incluídos (76,3%).

**Resultados:**

A mediana da idade foi 51,5 anos e a mediana do seguimento foi 102,7 meses. Um paciente era portador do vírus da imunodeficiência humana. Dor no membro foi o sintoma mais prevalente. O tempo médio entre os sintomas e o diagnóstico foi de 5,4 meses. O sítio anatômico mais afetado foram as vértebras (n = 16, 33,3%). Em relação à International Prognostic Index Score (IPI), 64,3% dos pacientes tinham baixo grau e 25,7% tinham baixo-intermediário. O diagnóstico histológico mais comum foi linfoma difuso de grandes células B (LDGCB), com 85,7% dos casos. A imunofenotipagem foi positiva para CD20 em 95,3%. Onze pacientes tinham fratura patológica. Todos receberam quimioterapia, sendo que 30% dos regimes incluíam rituximabe. A radioterapia foi utilizada em 38% dos pacientes. A porcentagem total de sobrevida foi de 50% e o tempo médio de sobrevida foi de 80 meses. A idade e o regime de quimioterapia juntos influenciaram diretamente a sobrevida dos pacientes. Os pacientes jovens, assim como os que receberam RCHOP, tiveram prognóstico de sobrevida melhor.

**Conclusão:**

A escolha do regime de quimioterapia associada à idade do paciente influenciou a sobrevida dos pacientes com LOP. Nível de Evidência IV; Série de casos.

## INTRODUCTION

Primary bone lymphoma (PBL) is rare cancer that accounts for 5% of non-Hodgkin’s lymphomas (NHL) and 3% of all malignant bone diseases.^1^ Most studies feature the same epidemiological characteristics, such as predominance in males, initial symptoms of the disease, diagnosis in the 5th and 6th decades of life, and relatively favorable survival prognosis.^1^ ^-^ ^4^ Even so, there is no consensus on best management because of the limited number of patients reported in the case series. There is also little agreement about treatment options and their results. The method of diagnosis often varies; more precise diagnosis allows better treatment choices. Streamlined staging has helped reduce the number of patients with PBL, excluding cases with evidence of systemic disease during the first six months of diagnosis.^5^ Treatment has been evolving since 1960, when radiation therapy was the method of choice, yielding high rates of local control of the disease but also high levels of systemic failure.^6^ ^-^ ^8^ Evidence of considerable increases in survival rates after adding chemotherapy to PBL treatment are frequent in the literature. Currently, treatment includes a combination of chemotherapy and radiation versus the traditional radiation alone.^4.9^ In the late 1990s, rituximab, a monoclonal anti-CD20 antibody, was added to chemotherapy and became the standard treatment for B-cell lymphomas. After rituximab was used for treatment, survival improved significantly for diffuse large B-cell lymphoma (DLBCL). Even so, the role of this drug in treating PBL is not yet clear because of the limited number of patients with this diagnosis. It is difficult to evaluate the benefits of rituximab in treating this disease because of its favorable prognosis for survival. An initial experience treating PBL in our service was published in 2002.^10^ At that time, our patients did not receive rituximab.^10^ We therefore resolved to review the cases of PBL diagnosed at our institution after 2002, to analyze how changes in diagnosis and treatment currently impact patients.

## MATERIALS AND METHODS

The study was approved by the HCFMUSP institutional review board (process 11313). This cross-sectional retrospective study utilized records from 55 patients diagnosed with PBL by the Orthopedic Oncology Group at IOT-HC-FMUSP from 2000 to 2016. Thirteen of the medical records were excluded due to lack of information, and 42 were included in the study. All the patients were diagnosed with PBL and received primary treatment. Age, sex, clinical stage (in accordance with the Ann Arbor system), serum lactic dehydrogenase level (LDH), presence of B symptoms, and IPI^11^ were acquired from the medical records. The histological diagnosis was performed or reviewed by pathologists from our institute, and the patients were classified according to the World Health Organization (WHO) classification for hematopoietic and lymphatic tissue tumors.^12^ Immunophenotype was identified with a immunohistochemical panel on paraffin-embedded tissue, using the following antibodies: BCL-2, BCL-6, CD3, CD5, CD10, CD15, CD20, CD30, CD45, CD79, and Ki-67. All patients received at least two cycles of rituximab to be considered for the medication use group. The clinical and demographic characteristics were described according to the following measures: average, standard deviation, median, minimum and maximum for quantitative variables, and absolutes and relatives for the qualitative variables.^13^ The average survival time estimated was according to characteristic using the Kaplan-Meier method.^14^ It was not possible to estimate the average survival for the entire cohort because the number of deaths was lower than 50%. Survival was compared between the categories using the log-rank test.^14^ The risk rate was estimated retrospectively (CI=95%) with bivariate Cox regression. The multiple Cox model was estimated using the variables that showed a descriptive level less than 0.2 (p<0.2), and only the variables with statistical significance remained in the final analysis. All tests were performed at a 5% significance level. The data were analyzed using Statistical Package for the Social Sciences software. This review follows our 2002 publication,^10^ and describes clinical and histological presentation, treatment, and results.

## RESULTS

**Figure 1:**
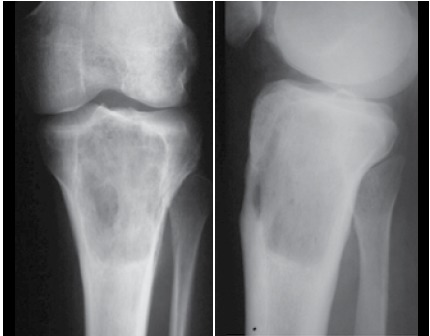
X-ray image of a primary bone lymphoma in the proximal tibia (prior to chemotherapy).

**Figure 2:**
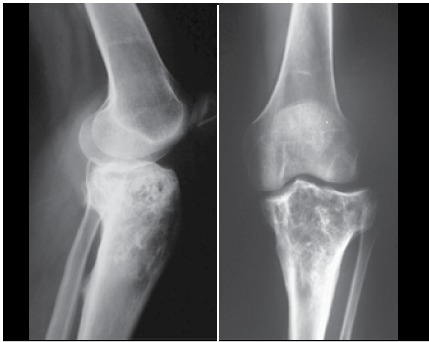
X-ray image of a primary bone lymphoma in the proximal tibia (after chemotherapy).

**Figure 3:**
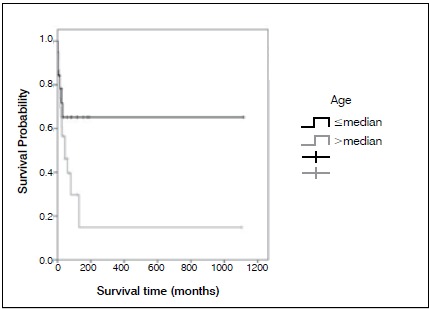
Average survival, according to age at diagnosis.

**Figure 4:**
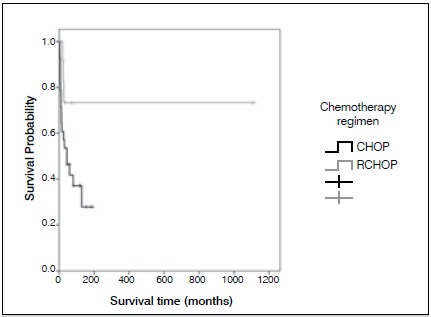
Average survival, according to chemotherapy regimen.

Most patients were men (61.9%), median age at diagnosis was 51.5 years, and median follow up was 102.7 months (from 4 to 114 months). One patient was HIV positive (2.4%). One patient had B symptoms (2.4%). Pain in the affected limb was the most prevalent symptom (100%). The average time between the appearance of symptoms and diagnosis was 5.4 months. The most affected areas were the vertebrae (n=16, 33.3%), the femur (n=12, 23.8%), and the tibia (n=4, 9.5%). LDH was elevated in 13 patients (30.9%). Eleven patients (26.2%) had pathological fracture at diagnosis. Twenty-three percent of patients were surgically treated, and arthrodesis was most commonly performed. Using the International Prognosis Index Score (IPI), 64.3% of patients were low grade and 25.7% were low-intermediate. The most common histological diagnosis was DLBCL, affecting 85.7% of patients. Immunophenotyping was performed in all cases, and 95.3% were CD20 positive. (Tables 1 and 2) All patients received chemotherapy, isolated, or in association with rituximab, and 38.1% of cases also received radiation therapy. Cases of pathologic fracture, imminent fracture, or medullary compression were treated surgically. Only two cases with pathological fracture did not receive surgical treatment. (Figures 1 and 2) The total percentage of survival was 50% and average survival was 80 months. The risk of death increased by 4% for each year of the patient’s age. Age and chemotherapy regimen together had a direct influence on patient survival. Young patients as well as those who received R-CHOP had a better prognosis for survival. It was not possible to estimate the average time for the entire cohort because the number of deaths was lower than 50%. (Figures 3 and 4)

**Table 1:** Population distribution according to characteristic.

Variable	Description (N=42)	
Sex, n (%)		
Female	16	(38.1)
Male	26	(61.9)
**Age (years)**		
Mean (SD)	49.4	(18.4)
Median (min-max)	51.5	(11-77)
**Location, n (%)**		
Vertebra	14	(33.3)
Femur	10	(23.8)
Tibia	4	(9.5)
Ilium	3	(7.1)
Humerus	3	(7.1)
Other	8	(19.0)
**Clinical presentation n (%)**		
Pain in affected site	42	(100.0)
Fever	1	(2.4)
**Duration of symptoms (months)**		
Mean (SD)	5.2	7
Median (min-max)	2	1 to 36
**LHD**		
Mean (SD)	834.9	954.3
Median (min-max)	549.5	168-593.4
**Pathological Fracture**		
No	31	73.8
Yes	11	26.2
**Histology**		
DLBCL	37	88
Diffuse small-cell lymphoma	2	4.8
Diffuse small-cell and large B-cell lymphoma	2	4.8
T-cell lymphoma	1	2.4
**IPI**		
Low	27	64.3
Low-intermediate	15	45.7

**Table 2:** Average survival according to data from comparative tests.

		CI	95%		CI	95%			
Variable	Mean Value	Below	Above	RR	Below	Above	Deaths	Total	%
	time (months)								
Age									
(<) / (=) Median	732.0	480.9	983.1	1.00			6	19	31.6
(>) Median	205.9	00.0	446.7	2.26	0.87	5.88	15	23	65.2
Sex									
Female	552.2	282.6	821.8	1.00			8	16	50.0
Male	408.5	144.2	672.9	1.00	0.41	2.40	13	26	50.0
Symptom duration									
(<) / (=) Median	229.8	00.0	491.7	1.00			15	23	65.2
(>) Median	748.3	507.4	989.3	0.44	0.17	1.14	6	19	31.6
HIV									
No	425.9	208.6	643.1	1.00			21	41	51.2
Yes	155.0	155.0	155.0	0.05	0.00	538.75	0	1	00.0
LHD									
Normal	453.0	133.8	772.2	1.00			8	17	47.1
Abnormal	461.6	214.0	709.3	0.85	0.35	2.07	13	25	52.0
Location									
Trunk	545.0	284.0	805.9	1.00			9	19	47.4
Limbs	444.2	190.3	698.1	0.91	0.38	2.18	12	23	52.2
Pathological Fracture									
No	490.0	238.0	741.9	1.00			14	31	45.2
Yes	83.7	35.3	132.0	1.46	0.59	3.63	7	11	63.6
Immunophenotyping									
CD3	71.5	55.6	87.4	1.00			1	2	50.0
CD20	68.2	41.3	95.0	1.84	0.24	14.24	13	22	59.1
Both	680.7	429.3	932.1	1.03	0.13	8.44	7	18	38.9
IPI									
Low	452.0	204.1	700.0	1.00			13	27	48.1
Low-Intermediate	505.4	216.5	794.2	1.29	0.53	3.13	8	15	53.3
Radiation therapy									
No	375.2	134.0	616.5	1.00			15	26	57.5
Yes	681.4	415.6	947.2	0.74	0.28	1.91	6	16	37.5
Chemotherapy									
CHOP	80.6	50.5	110.7	1.00			18	28	64.3
R-CHOP	824.0	541.0	1107.1	0.31	0.09	1.06	3	14	21.4
Total	455.8	249.2	662.5				21	42	50.0

## DISCUSSION

The medical literature does not feature an extensive volume of publications on PBL, mainly because of the rarity of this disease. Most of the lymphoma cases actually presented extra-osseous disease. Therefore, most of the published studies are retrospective series. The present study was no exception; we analyzed 55 consecutive cases over 15 years, 13 of which were excluded due to lack of complete information in the medical records. In our previous study from 2002,^10^ 57 of the 81 cases were excluded due to lack of data in the patient chart. Comparatively, in the larger published series, we found one study involving 119 cases over 38 years, and in another 82 cases over 40 years.^15^ ^,^ ^16^ This makes the present study the largest series reported in Latin America. We also observed that our service had twice the number of cases of the other studies in half the time, which reflects improvement in the ability to diagnose patients with PBL. The average amount of time from appearance of symptoms to date of diagnosis was 5.4 months, similar to the data reported in other studies.^16^ This also shows the improved access to public health that residents in some areas of Brazil currently enjoy. However, and particularly in our study, time of diagnosis did not affect the prognosis of patients with PBL. As expected, the epidemiological characteristics of PBL were found in our series: the median age was in the fifties and sixties, men were more prevalent, and pain was the main symptom. An important difference from the literature is that the most frequently affected site was the vertebrae, as we also found in our previous publication.^10^ Other studies generally show that the femur is most frequently affected.^15^ ^,^ ^16^ Few prognostic factors were found in the statistical analysis. In our study, age appears to be a prognostic factor, which is compatible with the literature;^4^ ^,^ ^16^ younger patients had a more favorable prognosis for survival. There is no consensus on the significance of the prognosis and location of disease involvement (axial or appendicular). We also did not find a statistically significant difference in site affected in relation to survival prognosis in our series.^3^ ^,^ ^10^ ^,^ ^17^ The IPI scale is not used universally for reporting PBL, but in our study we found that most patients were classified as low grade, which is similar to the findings of publications that use this same scale.^15^ The fact that the majority of publications do not use the IPI shows that this scale does not play a definitive role in the prognosis for PBL. In terms of treatment, chemotherapy is always used to treat PBL, even when the disease is localized.^5^ ^,^ ^16^ We also found that rituximab, a monoclonal antibody directed against B-cell marker CD20, which is expressed in NHL, has been used to treat bone lymphoma since 1997.^17^ ^,^ ^18^ However, this medication has only been approved for use in the Brazilian public health system since 2012. Consequently, only 13 patients (30%) in our series received this medication, which may have had a negative impact on survival prognosis in our cases. In the present study, lower age as well as the use of rituximab were significantly associated with better survival prognosis in patients with PBL. Because it is a rare disease, compilation of a large number of PBL cases is a lengthy process, which leads to a bias in relation to the diagnosis and treatment of this disease, in turn compromising analysis of the prognosis in the patients studied. In comparison with our previous study,^10^ we observed significant differences in relation to current diagnosis and treatment of PBL. For example, in the previous study fewer than 80% of the patients received chemotherapy as part of their treatment; in our current study, 100% of patients were treated with chemotherapy. We can conclude that chemotherapy should continue to be used in all cases of PBL, with the addition of radiotherapy and surgery in specific situations. We should also mention that immunotherapy with rituximab should be part of chemotherapy treatment for a better survival prognosis in patients affected by this disease.

## CONCLUSION

The use of rituximab in the treatment regimen and lower patient age were associated with better survival prognosis in patients with PBL.

## References

[1] Baar J, Burkes RL, Gospodarowicz M (1999). Primary non-Hodgkin&apos;s lymphoma of bone. Semin Oncol.

[2] Barbieri E, Cammelli S, Mauro F, Perini F, Cazzola A, Neri S (2004). Primary non-Hodgkin&apos;s lymphoma of the bone treatment and analysis of prognostic factors for Stage I and Stage II. Int J Radiat Oncol Biol Phys.

[3] Sutcliffe SB, Gospodarowicz MK, Bush RS, Brown TC, Chua T, Bean HA (1985). Role of radiation therapy in localized non-Hodgkin&apos;s lymphoma. Radiother Oncol.

[4] Zinzani PL, Carrillo G, Ascani S, Barbieri E, Tani M, Paulli M (2003). Primary bone lymphoma experience with 52 patients. Haematologica.

[5] Takahashi H, Tomita N, Yokoyama M, Tsunoda S, Yano T, Murayama K (2012). Prognostic impact of extranodal involvement in diffuse large B-cell lymphoma in the rituximab era. Cancer.

[6] Wang CC, Fleischli DJ (1968). Primary reticulum cell sarcoma of bone With emphasis on radiation therapy. Cancer.

[7] Boston HC, Dahlin DC, Ivins JC, Cupps RE (1974). Malignant lymphoma (so-called reticulum cell sarcoma) of bone. Cancer.

[8] Dosoretz DE, Murphy GF, Raymond AK, Doppke KP, Schiller AL, Wang CC (1983). Radiation therapy for primary lymphoma of bone. Cancer.

[9] Fidias P, Spiro I, Sobczak ML, Nielsen GP, Ruffolo EF, Mankin H (1998). Long-term results of combined modality therapy in primary bone lymphomas. Int J Radiat Oncol Biol Phys.

[10] de Camargo OP, dos Santos Machado TM, Croci AT, de Oliveira CR, Giannotti MA, Baptista AM (2002). Primary bone lymphoma in 24 patients treated between 1955 and 1999. Clin Orthop Relat Res.

[11] International Non-Hodgkin&apos;s Lymphoma Prognostic Factors Project (1993). A predictive model for aggressive non-Hodgkin&apos;s lymphoma. N Engl J Med.

[12] Swerdlow SH (2008). World Health Organization classification of tumors of haematopoietic and lymphoid tissues.

[13] Kirkwood BR, Sterne JA (2006). Essential medical statistics.

[14] Kleinbaum DG (1996). Survival analysis: a self-learning text.

[15] Beal K, Allen L, Yahalom J (2006). Primary bone lymphoma treatment results and prognostic factors with long-term follow-up of 82 patients. Cancer.

[16] Demircay E, Hornicek FJ, Mankin HJ, Degroot 3rd H (2013). Malignant lymphoma of bone a review of 119 patients. Clin Orthop Relat Res.

[17] Horsman JM, Thomas J, Hough R, Hancock BW (2006). Primary bone lymphoma a retrospective analysis. Int J Oncol.

[18] Scott SD (1998). Rituximab a new therapeutic monoclonal antibody for non-Hodgkin&apos;s lymphoma. Cancer Pract.

